# Quantification of Coumarins in Aqueous Extract of *Pterocaulon balansae* (Asteraceae) and Characterization of a New Compound

**DOI:** 10.3390/molecules201018083

**Published:** 2015-10-02

**Authors:** Bruna Medeiros-Neves, Francisco Maikon Corrêa de Barros, Gilsane Lino von Poser, Helder Ferreira Teixeira

**Affiliations:** Programa de Pós-Graduação em Ciências Farmacêuticas, Faculdade de Farmácia, Universidade Federal do Rio Grande do Sul, Avenida Ipiranga, 2752, Porto Alegre, RS 90610-000, Brazil; E-Mails: farmbrunamedeiros@gmail.com (B.M.-N.); barrosfarm@yahoo.com.br (F.M.C.B.); gilsane@farmacia.ufrgs.br (G.L.P.)

**Keywords:** coumarin, *Pterocaulon balansae*, HPLC, UPLC-UV-MS, 5-methoxy-6,7-methylenedioxycoumarin, 5,6-dimethoxy-7-(2′,3′-epoxy-3′-methylbutyloxy)coumarin

## Abstract

Plants from the genus *Pterocaulon* are popularly used as antifungal and wound-healing agents. Such activities have been related to coumarins, which are abundant in those plants. Coumarins are soluble in organic solvents, such as hexane and dichloromethane, and some of them are also soluble in hot water. Considering that infusion and decoctions of these plants are used in traditional medicine, the aim of this study was to identify and quantify the coumarins in the aqueous extract of *Pterocaulon balansae.* The aqueous extract was obtained by dynamic maceration and the compounds were characterized by UPLC-UV-MS analysis. A new coumarin and 5-methoxy-6,7-methylenedioxycoumarin, used for validation of the analytical HPLC method were obtained by partition of the aqueous extract with *n*-hexane. The HPLC method validated was linear, specific, and precise. Seven coumarins were characterized in the aqueous extract in a range of 0.584–54 mg/g of dry plant material. The main compound, 5,6-dimethoxy-7-(3′-methyl-2′,3′-dihydroxybutyloxy)coumarin, is described for the first time in *P. balansae* together with a new compound, 5,6-dimethoxy-7-(2′,3′-epoxy-3′-methylbutyloxy)coumarin.

## 1. Introduction

Extraction and fractions obtained from medicinal plants have been investigated aiming to isolate compounds with potential pharmacological activities [1]. An ethnoveterinary survey indicated that plants from the genus *Pterocaulon* (Asteraceae) are used in Brazilian traditional medicine to treat skin diseases caused by both bacteria and fungi [2]. In recent years, various pharmacological studies conducted on the extracts of these plants have supported their ethnomedical use and the biological properties are attributed to coumarins, the most abundant compounds found in these species [3,4,5].

Among the coumarins present in *Pterocaulon* species, 5-methoxy-6,7-methylenedioxycoumarin, identified in *P. alopecuroides*, *P. balansae*, *P. polystachyum*, *P. redolens*, *P. serrulatum*, and *P. virgatum* [6,7,8,9,10], is the most studied. This compound demonstrated cytotoxicity against glioma cell lines and was able to induce markers of cell differentiation, suggesting a potential therapeutic application in the management of leukemia [11,12].

Coumarins are very soluble in chloroform, diethyl ether, pyridine, and slightly soluble in ethanol and water (100 mg/L at 25 °C). Nevertheless, some of them are soluble in hot water [13,14]. In previous studies carried out with *P. balansae* the coumarins were extracted by either Soxlet or maceration using *n*-hexane, dichloromethane, methanol chloroform or ethyl acetate [3,15,16,17,18].

Considering that aqueous extracts, both infusion and decoctions of these plants are the main preparations used in traditional medicine, the aim of this study was to identify the coumarins present in the aqueous extract of *P. balansae* by UPLC-UV-MS and to develop and validate an HPLC method for their quantification.

## 2. Results and Discussion

### 2.1. Extraction and Isolation

**Figure 1:**
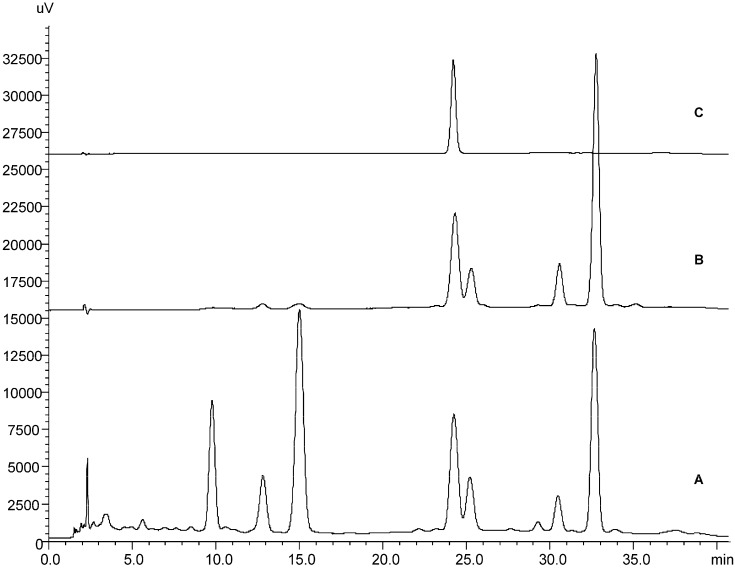
HPLC chromatograms of (**A**) aqueous extract (**B**) *n*-hexane fraction; and (**C**) 5-methoxy-6,7-methylenedioxycoumarin isolated from *P. balansae*.

Despite the lipophilicity of the coumarins, it is well established that some of them can be extracted using hot water [14], one of the most suitable vehicles for the preparation of phytopharmaceuticals [19]. In a preliminary study, aqueous extracts of *P. balansae* were obtained by using different temperatures (25 °C, 40 °C, and 60 °C). These extracts were analyzed by HPLC-DAD and all of them presented the same chromatographic profile, displaying seven main peaks with typical ultraviolet absorption spectra of coumarin derivatives (unpublished data). The extract obtained at 60 °C was selected for further studies due to the high content of coumarins. The aqueous extract was partitioned by liquid-liquid extraction to produce the *n*-hexane fraction, which presented four main compounds. Addition of methanol in the dry *n*-hexane fraction afforded 5-methoxy-6,7-methylenedioxycoumarin. This compound was identified by comparison of HPLC retention time with a standard sample previously isolated from the same plant [10] and by spectroscopic analysis. The whole process is represented in the chromatograms shown in Figure 1.

### 2.2. UPLC-UV-MS Analysis

**Figure 2:**
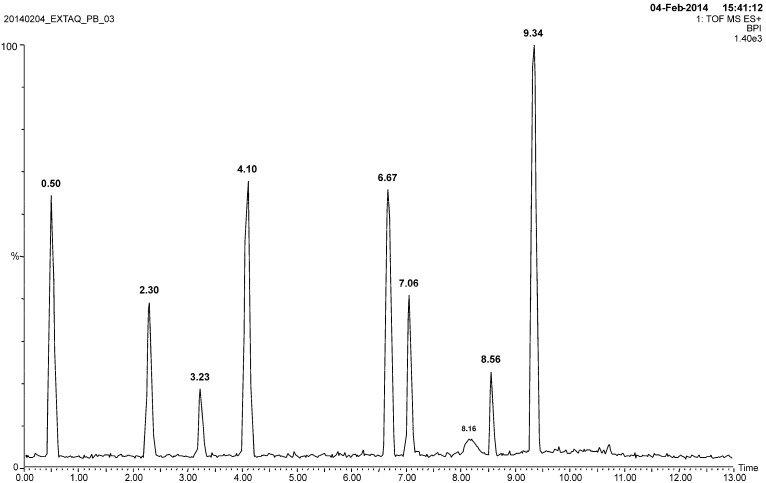
Chromatographic profile of the aqueous extract by UPLC method.

The UPLC profile of the aqueous extract of *P. balansae* is shown in Figure 2. The peaks were characterized by a combination of UV and ESI-MS analysis. Table 1 presents the maximum UV absorptions and molecular ions [M + H]^+^ from UPLC-UV and UPLC-MS, respectively.

The coumarins 7-(2′,3’′-dihydroxy-3′-methylbutyloxy)-6-methoxycoumarin (**1**), 5-(2′,3′-dihydroxy-3′-methylbutyloxy)-6,7-methylenedioxycoumarin (**2**), 5-methoxy-6,7-methylenedioxycoumarin(**4**), 7-(2′,3′-epoxy-3′-methyl-3′-butyloxy)-6-methoxycoumarin (**5**) and 5-(2′,3′-epoxy-3′-methylbutyloxy)-6,7-methylenedioxycoumarin (**6**) were previously described for *P. balansae* [10,16,20,21]. The compounds were characterized by their molecular ion peaks and fragmentation data and by UV absorption as well as by comparison of their HPLC retention times with published data. These data are in accordance with those found in literature [16,20,21].

As far is known, this is the first time that 5,6-dimethoxy-7-(3′-methyl-2′,3′-dihydroxybutyloxy)coumarin (**3**) was found in *P. balansae*, while 5,6-dimethoxy-7-(2′,3′-epoxy-3′-methylbutyloxy)coumarin (**7**) was identified as a new compound. An isomer of this coumarin was previously isolated from the same plant by Magalhães and co-workers (1981). According to the authors, the isomer of compound **7** has no oxygen at the position 5, once the signal assigned to H-4 is at relatively high field in the ^1^H-NMR [15]. Contrarily, in the present study, the signal of H-4 appears as a doublet at 7.92 ppm, indicating the presence of an oxygenated function in the position 5, as discussed below.

**Table 1:** Relationship between the absorption maxima in the UV and maximum abundance in the ESI-MS for the compounds of the aqueous extract of *P. balansae*.

Compound	Structure	Molecular Formula	Time Retention (min)	Max. Absorption (nm)	100% Abundance (*m*/*z*)
**1**	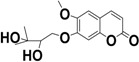	C_15_O_6_H_18_	2.30	229, 293, 343	295.1319
**2**	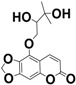	C_15_O_7_H_15_	3.23	239, 327	309.1142
**3**	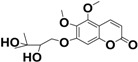	C_16_O_7_H_20_	4.10	228, 327	325.1426
**4**	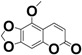	C_11_O_5_H_8_	6.67	240, 325	221.0555
**5**	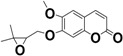	C_15_O_5_H_16_	7.06	236, 292, 341	277.1207
**6**	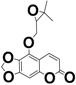	C_15_O_6_H_14_	8.56	243, 324	291.1016
**7**	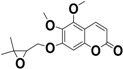	C_16_O_6_H_18_	9.34	235, 325	307.1302

### 2.3. Structural Elucidation of 5,6-Dimethoxy-7-(2′,3′-epoxy-3′-methylbutyloxy)coumarin

The structure of the new coumarin isolated from *P. balansae* was elucidated by the spectral analysis consisting of ^1^H-NMR, COSY, ^13^C-NMR, DEPT, HMQC, HMBC, and ESI-MS.

Compound **7** was isolated as a yellowish amorphous powder. The molecular formula was determined as C_16_O_6_H_18_ on the basis of the HR-ESI-MS (*m*/*z* 307.1302, calculated 307.1182 [M + H]^+^). The UV spectrum showed an absorption band with λ_max_ 235 and 325 nm, characteristic of 5,6,7-trioxygenated coumarins which present the λ_max_ at 325 to 330 nm [22].

The ^1^H-NMR spectrum (Table 2) showed a pair of doublets at δ_H_ 6.23 (d, *J* = 9.6 Hz) and δ_H_ 7.92 (d, *J* = 9.6 Hz) relative to the conjugated olefinic protons H-3 and H-4, respectively, characteristic of coumarins. The chemical shift of H-4 at δ_H_ 7.92 is consistent with the presence of oxygen function at the C-5 position [23]. Oxygenated substituent at this position shifts the proton at C-4 to lower fields, around 8 ppm [15].

**Table 2:** ^1^H- (400 MHz) and ^13^C- (100 MHz) NMR data of 5,6-dimethoxy-7-(2′,3′-epoxy-3′-methylbutyloxy)coumarin (CDCl_3_).

Position	δ_C_ ppm	δ_H_ ppm (*J* in Hz)
**2**	161.16	-
**3**	112.71	6.23 (d, *J* = 9.6, 1H)
**4**	138.78	7.92 (d, *J* = 9.6, 1H)
**5**	149.41	-
**6**	138.27	-
**7**	151.25	-
**8**	96.62	6.64 (s, 1H)
**9**	156.03	-
**10**	107.55	-
**1′b**	68.42	4.30 (dd, *J* = 11.1; 3.6, 1H)
**1′a**		4.09 (dd, *J* = 11.0; 6.4, 1H)
**2′**	61.25	3.20 (dd, *J* = 5.7; 3.8, 1H)
**3′**	58.21	-
**4′**	19.08	1.39 (s, 3H)
**5′**	24.55	1.40 (s, 3H)
**5-OMe**	61.84	4.03 (s, 3H)
**6-OMe**	60.80	3.88 (s, 3H)

The singlets at δ_H_ 4.03 (3H) and δ_H_ 3.88 (3H) were assigned to the methoxy groups attached to C-5 and C-6, respectively. The singlet at δ_H_ 6.64 was assigned to H-8. At δ_H_ 4.30 (*J* = 11.1; 3.6 Hz), δ_H_ 4.09 (*J* = 11.0; 6.4 Hz), and δ_H_ 3.20 (*J* = 5.7; 3.8 Hz) it was observed three broad double doublets with integration of one proton to each signal. These results indicated the presence of an ABX type system in which A and B were assigned to non-equivalent geminal protons (H-1′b and H-1′a, respectively) and X attributed to the epoxide proton (H-2′) (Table 2) [20]. Additionally, the chemical shifts of these protons are compatible with the presence of oxygenated substituent at the C-7 position [20]. The singlets at δ_H_ 1.40 and δ_H_ 1.39 (3H each) indicated the presence of geminal dimethyl group at C-4' and C-5', respectively [20].

Perusal of the ^13^C-NMR spectra, both 1D (Table 2) and 2D-(APT, HSQC, and HMBC) (Supplementary Material), permitted to characterize all carbons of the molecule. Seven quaternary carbons were identified (δ_C_ 161.16, δ_C_ 156.03, δ_C_ 151.25, δ_C_ 149.41, δ_C_ 138.27, δ_C_ 107.55, δ_C_ 58.21). The δ_C_ 161.16 was attributed to C-2 carbon of the carbonyl lactonic that should be less protected. The tertiary, secondary, and primary carbons were identified using the data provided by the spectra of APT, HSQC, HMBC correlating with the data already obtained for the hydrogens of the molecule. The presence of oxygen function at the C-5 position was confirmed by the HMBC spectrum. The correlation observed between the 3H-singlet (δ_H_ 4.03) and the aromatic carbon (δ_C_ 149.41), indicates that the methoxy group is attached to C-5. The presence of the other methoxy group attached to C-6 was also confirmed by the HMBC correlation. The NMR signals are very similar to those published for another 5,6,7-trioxygenated coumarins presenting methoxy group at C-5 and C-6 [24].

### 2.4. Validation of an HPLC Method for 5-Methoxy-6,7-methylenedioxycoumarin

The linearity of the method was assessed by means of the concentration of 5-methoxy-6,7-methylenedioxycoumarin *vs.* the corresponding mean peak area. The standard curve for quantification of 5-methoxy-6,7-methylenedioxycoumarin was obtained in a concentration range between 0.5 and 10 µg/mL. The equation of the straight line obtained by linear regression by the least squares method was y = 54389x + 1563.5. The coefficient of determination (R^2^) obtained was 0.9991, which demonstrates the high correlation between the concentration of 5-methoxy-6,7-methylenedioxycoumarin and peak area. Thus, the results demonstrate that the method is linear within the concentration range specified, showing a significant linear regression (*p* < 0.05).

The sensitivity of the method was evaluated by determining the limits of detection (LOD) and quantification (LOQ). These determinations were based on standard deviation of the response and slope of the calibration. The LOD and LOQ were 0.062 and 0.187 μg/mL, respectively, demonstrating satisfactory sensitivity of the method.

The accuracy of the method was evaluated at three levels: 0.5, 4.0, and 7.0 μg/mL for 5-methoxy-6,7-methylenedioxycoumarin, with recoveries of approximately 100.0%, without statistical difference by the F test (*p* > 0.05) and RSD < 2.0% in all determinations of the recovery test. The method showed good accuracy at low, medium, and high level concentrations for 5-methoxy-6,7-methylenedioxycoumarin, within the linearity of the method. This validation parameter shows good reliability for determining the level of 5-methoxy-6,7-methylenedioxycoumarin in the aqueous extract of *P. balansae*.

The mean, standard deviation, RSD and 95% confidence interval at all concentration levels were calculated for the three days. The results showed RSD less than 5% for experiments on the same day or three different days, indicating that the method is precise, according to the official codes (ICH, 2013).

### 2.5. Quantification of the Coumarins in P. balansae Aqueous Extract

The amount of coumarins present in the aqueous extract was determined using the HPLC method and the results are expressed in terms of 5-methoxy-6,7-methylenedioxycoumarin (Table 3).

**Table 3:** Coumarin contents in the aqueous extract of *P. balansae*.

Coumarins	mg/g Dried Plant Expressed in 5-Methoxy-6,7-methylenedioxycoumarin
**1**	1.90
**2**	1.02
**3**	4.54
**4**	2.33
**5**	1.01
**6**	0.58
**7**	3.33

According to the results, it can be observed that the main compounds present in this extract are the compound **3**, followed by the compound **7**. It is interesting to observe that the more lipophilic compounds (coumarins **5**, **6** and **7**), contain an epoxy group and can be the precursors of the more polar compounds (coumarins **1**, **2** and **3**, respectively) by the opening of the epoxy ring.

## 3. Experimental Section

### 3.1. Plant Material

Aerial parts of *Pterocaulon balansae* Chodat were collected in Canoas, RS, Brazil, in February 2013. The species was identified by Dr. Sérgio A. L. Bordignon (Centro Universitário La Salle-Canoas, Unilasalle, Brazil). Voucher specimen was deposited in the herbarium of the Universidade Federal do Rio Grande do Sul (ICN 157762). Plant collection was authorized by Ministério do Meio Ambiente—MMA, Instituto Chico Mendes de Conservação da Biodiversidade—ICMBio. Sistema de Autorização e Informação em Biodiversidade-SISBIO (number 38017-1).

### 3.2. Solvents

The solvent *n*-hexane was obtained from Synth (Synth, Diadema, Brazil), acetic acid, ethanol and methanol from Nuclear (Nuclear, Diadema, Brazil), deuterated chloroform from Merck^®^ (Merck, Darmstadt, Germany) and HPLC grade acetonitrile and formic acid from Tedia (Tedia, Fairfield, OH, USA). Ultrapure water (H_2_O) was obtained from a Milli-Q^®^ Plus apparatus by Millipore (Millipore, Billerica, MA, USA).

### 3.3. Nuclear Magnetic Resonance (NMR) Analysis

1D-(^1^H and ^13^C) and 2D-(APT, HSQC, HMBC, COSY) NMR spectra were recorded on a Varian MR400 spectrometer (^1^H at 400 MHz and ^13^C at 100 MHz, Agilent Technologies, Palo Alto, CA, USA) and the compounds were dissolved in CDCl_3_.

### 3.4. Extraction and Isolation

A preliminary screening of aqueous extraction was carried out with plant material and water (1:30 (*w*/*v*)) at different temperature (25 °C, 40 °C, and 60 °C) in the multipoint stirrer with water bath (Dist-DI920) for 1 h. The temperature 60 °C was selected to obtain the extract.

#### 3.4.1. Isolation of 5-Methoxy-6,7-methylenedioxycoumarin

The aqueous extract was filtered and the volume reduced by vacuum evaporation. This aqueous extract was subjected to liquid-liquid partition with *n*-hexane (10×). The organic phases were reunited and evaporated to dryness under reduced pressure at 45°C. Addition of methanol into the residue resulted in the formation of an insoluble precipitate which consisted of pure 5-methoxy-6,7-methylenedioxycoumarin. The compound was identified by comparison of HPLC retention time with a standard sample and by ^1^H- and ^13^C-NMR spectroscopy and the data were compared to those previously published by STEIN *et al.* (2007) [10]. The methanol soluble fraction was submitted to chromatographic methods described below.

#### 3.4.2. Isolation of 5,6-Dimethoxy-7-(2′,3′-epoxy-3-methylbutyloxy) Coumarin

The isolation of the compound from the methanol soluble fraction was carried out by preparative thin layer chromatography (TLC) using silica gel60 (0.20 mm, Merck, Darmstadt, Germany) pre-coated glass plates as stationary phase and dichloromethane:methanol (98:2) as mobile phase. After migration, the plates were visually inspected under UV light at 254 nm or 366 nm. The most intense band was scraped off the plate, and the obtained silica was eluted three times with dichloromethane affording the purified compound which was identified through spectroscopic methods (one-dimensional (^1^H and ^13^C) and two-dimensional (COSY, HSQC and HMBC) NMR as well as high resolution ESI/MS analysis (Waters, Milford, MA, USA).

### 3.5. HPLC Analysis

The samples were analyzed by high performance liquid chromatography (HPLC), equipped with a UV detector (Shimadzu, Kyoto, Japan). Phenomenex-C_18_ Synergi column (150 mm × 4.6 mm, 4 µm) coupled to a refillable pre-column filled with C_18_ silica was used in the analysis. The mobile phase consisted of a gradient of acetic acid 2% (A) and acetonitrile (B) filtered and degassed. The gradient elution was 17% B in 0.01 min, 17%–20% B in 10 min, 20% B in 15 min, 20%–25% B in 20 min, 25%–27% B in 22 min, 27%–30% B in 25 min, 30%–35% B in 30 min, 35% B in 35 min, 35%–17% B in 40 min. LC system operated at flow rate of 1 mL/min for 45 min at 30°C with the injection volume of 20 µL and the wavelength was adjusted to 327 nm.

### 3.6. UPLC-UV-MS Analysis

The aqueous extract was also analyzed by ultra-performance liquid chromatography (UPLC) coupled with ultraviolet (UV) and mass spectrometer (MS/MS) detector. The analysis was performed on a Micromass-LCT Premier Time of Flight (TOF) mass spectrometer (Waters, Milford, MA, USA) with an electrospray interface and coupled with an Acquity UPLC system (Waters). ESI conditions: capillary voltage 3000 V, sample cone 30 V, source temperature 120 °C, desolvation temperature of 300 °C, cone gas flow 70 L/h and desolvation gas flow of 350 L/h. Detection was performed in positive ion mode in the m/z range 50–1000.

The HPLC method above described was successfully transferred to UPLC with aid of ACQUITY UPLC^®^ Columns calculator (Waters). The separation was carried out on Phenomenex Kinetex-C18 UPLC columns at 40°C (C18: 100 mm × 2.10 mm, pore size 1.7 μm, 100 Å) with the following solvent system: A = 0.1 vol % formic acid-water, B = acetonitrile. The gradient elution was 17% B in 0.03 min, 17%–20% B in 2.86 min, 20% B in 4.28 min, 20%–25% B in 5.70 min, 25%–27% B in 6.26 min, 27%–30% B in 7.11 min, 30%–35% B in 8.53 min, 35% B in 9.95 min, 35%–17% B in 11.36 min. The LC system operated at flow rate of 490 μL/min for 13 min.

### 3.7. Quantification of Coumarins

For the quantification of the coumarins present in the aqueous extract, the above described HPLC method was validated according to an official compendium [25] using 5-methoxy-6,7-methylenedioxycoumarin as a reference compound. The parameters specificity, linearity, limit of detection, limit of quantification, accuracy, and precision were determined. The results were evaluated by means of an ANOVA single factor.

#### 3.7.1. Linearity

To verify the linearity of the method, 5-methoxy-6,7-methylenedioxycoumarin was solubilized in dimethyl sulphoxide (DMSO) and adjusted to the concentration of 40 µg/mL with acetonitrile and diluted with mobile phase ACN:H_2_O (1:1) to get concentration range of 0.5–10 µg/mL. These solutions were injected in the HPLC equipment in triplicate on three different days.

#### 3.7.2. Detection Limit and Quantification Limit

The detection and quantification limits (LOD and LOQ, respectively) were calculated from the calibration curve using the values of standard deviation of the intercept (σ) and of the slope (S) (LOD 3.3 σ/S and LOQ 10 σ/S).

#### 3.7.3. Accuracy

To determine the accuracy, the aqueous extract was prepared and spiked with known amounts of analyte, at the three concentrations of the 5-methoxy-6,7-methylenedioxycoumarin solution: low, medium, and high, corresponding to 0.5, 4.0, and 7.0 µg/mL, respectively, performing five determinations for each concentration. The results represent the mean recovery (%) for three independent samples.

#### 3.7.4. Precision

The intra-day precision (repeatability) of the method was determined by analysis of five determinations of 5-methoxy-6,7-methylenedioxycoumarin in three points of the analytical curves, during the same day under the same experimental conditions. Inter-day precision values were obtained by assaying freshly prepared solutions as 5-methoxy-6,7-methylenedioxycoumarin analytical curve on three different days. The results were expressed in relative standard deviation (RSD).

## 4. Conclusions

In the present study, it was demonstrated that the coumarins from *P. balansae* were efficiently obtained in aqueous extract. The compound 5-methoxy-6,7-methylenedioxycoumarin was achieved in satisfactory amounts and the developed analytical method was linear, specific and precise. Among the coumarins determined in the extract, **3** was not previously reported for this species. Additionally, the coumarin **7** was characterized as a new compound, which contributes to the chemical knowledge of *Pterocaulon* genus.

## Supplementary Material

Supplementary material including HMBC, HSQC, APT, COSY and^1^H- and ^13^C-NMR spectra of compound **7**, HRMS and UV spectra of compound **1**–**7**. Supplementary materials can be accessed at: http://www.mdpi.com/1420-3049/20/10/18083/s1.
