# Supplementary material for: Quantification of Coumarins in Aqueous Extract of Pterocaulon balansae (Asteraceae) and Characterization of a New Compound
Source: Molecules. 2015 Oct 2;20(10):18083–94. doi: 10.3390/molecules201018083 (PMC6331981; doi:10.3390/molecules201018083)
Supplement: Supplementary file 1 [file molecules-20-18083-s001.pdf]

Supplementary Material

Compound **7**

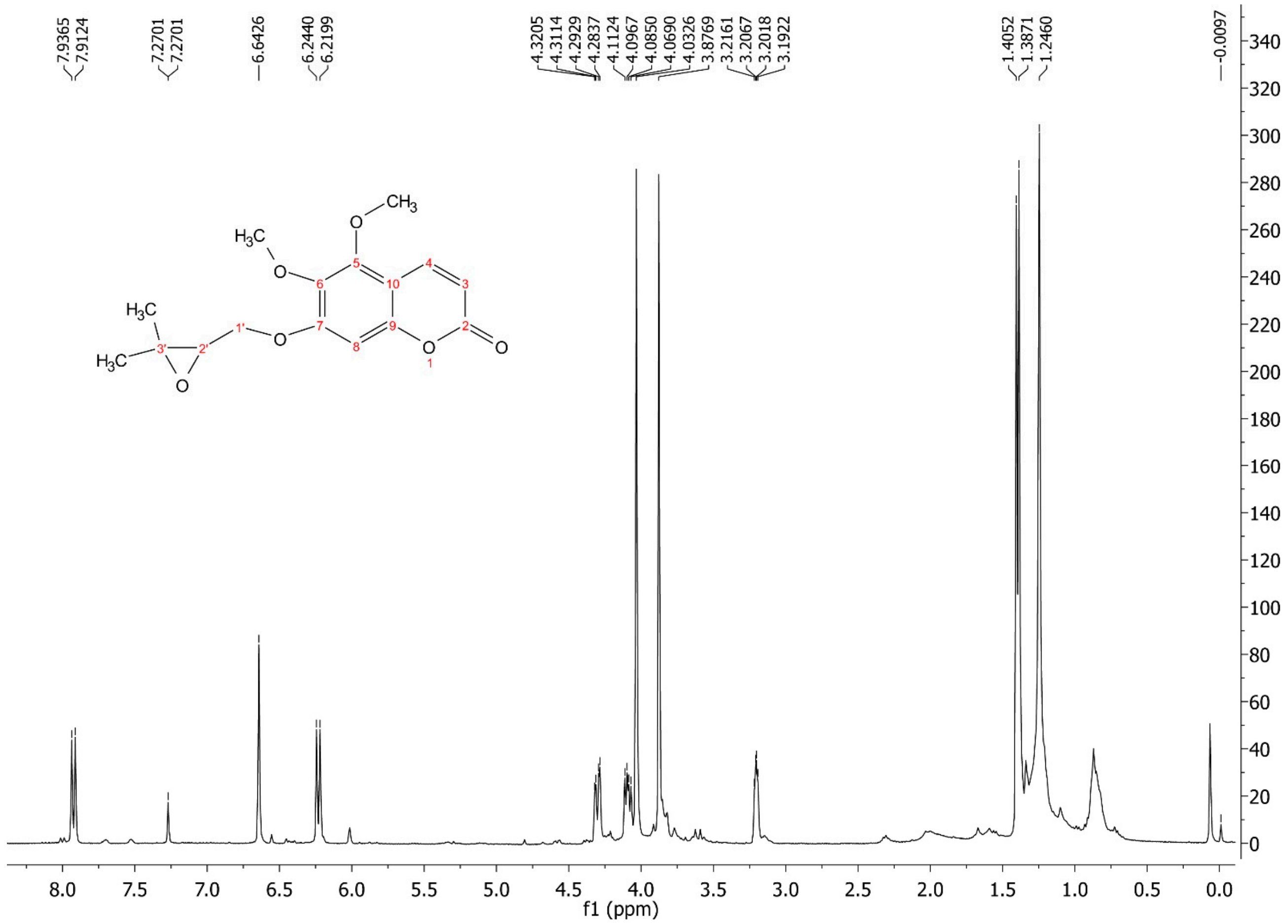

Figure S1. ^{1}H-NMR spectrum of 5,6-dimethoxy-7-(2′,3′-epoxy-3′-methylbutyloxy)coumarin (**7**) ($CDCl_3$, 400 MHz).

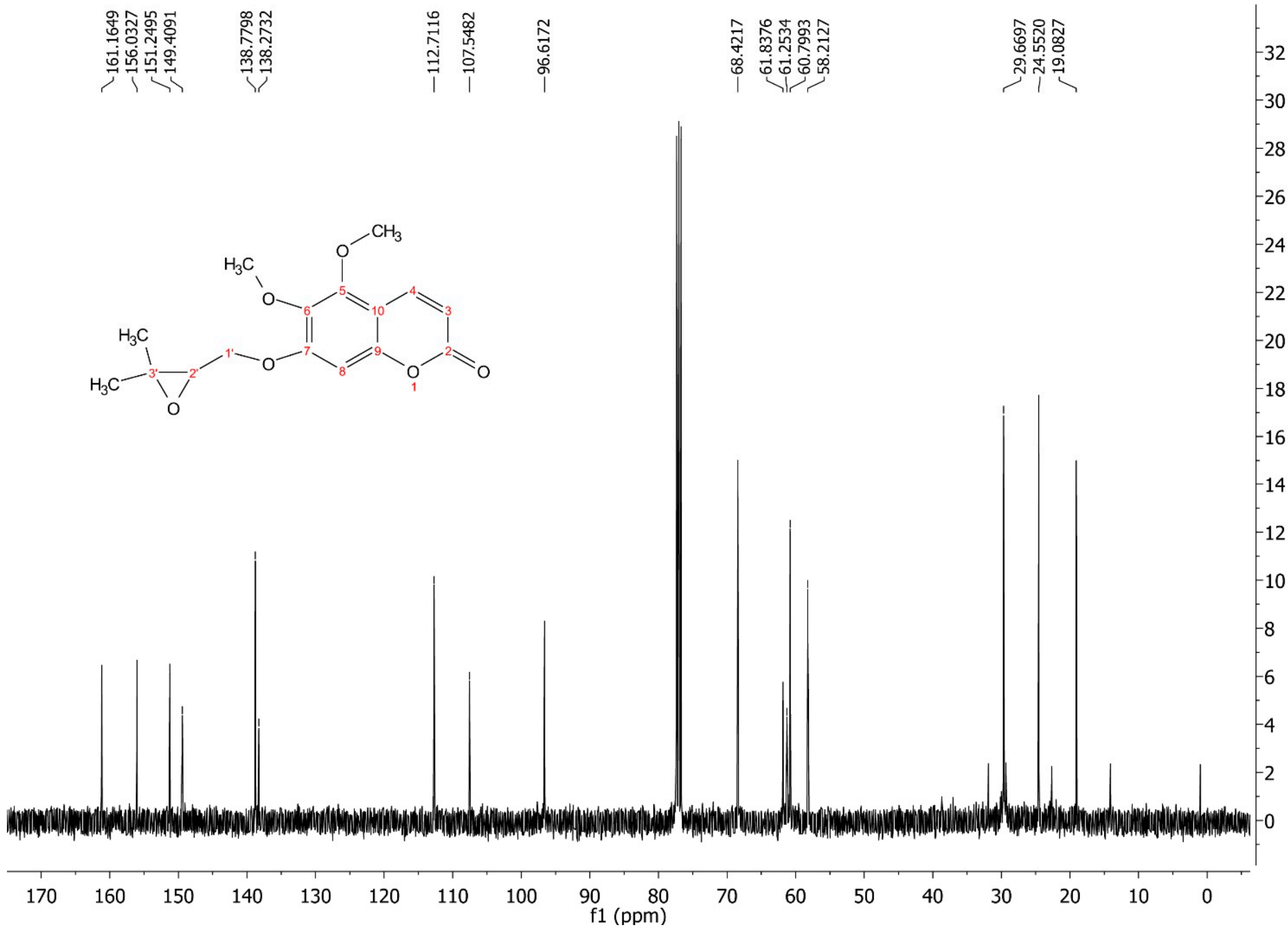

Figure S2. ^{13}C-NMR spectrum of 5,6-dimethoxy-7-(2′,3′-epoxy-3′-methylbutyloxy)coumarin (**7**) ($CDCl_3$, 100 MHz).

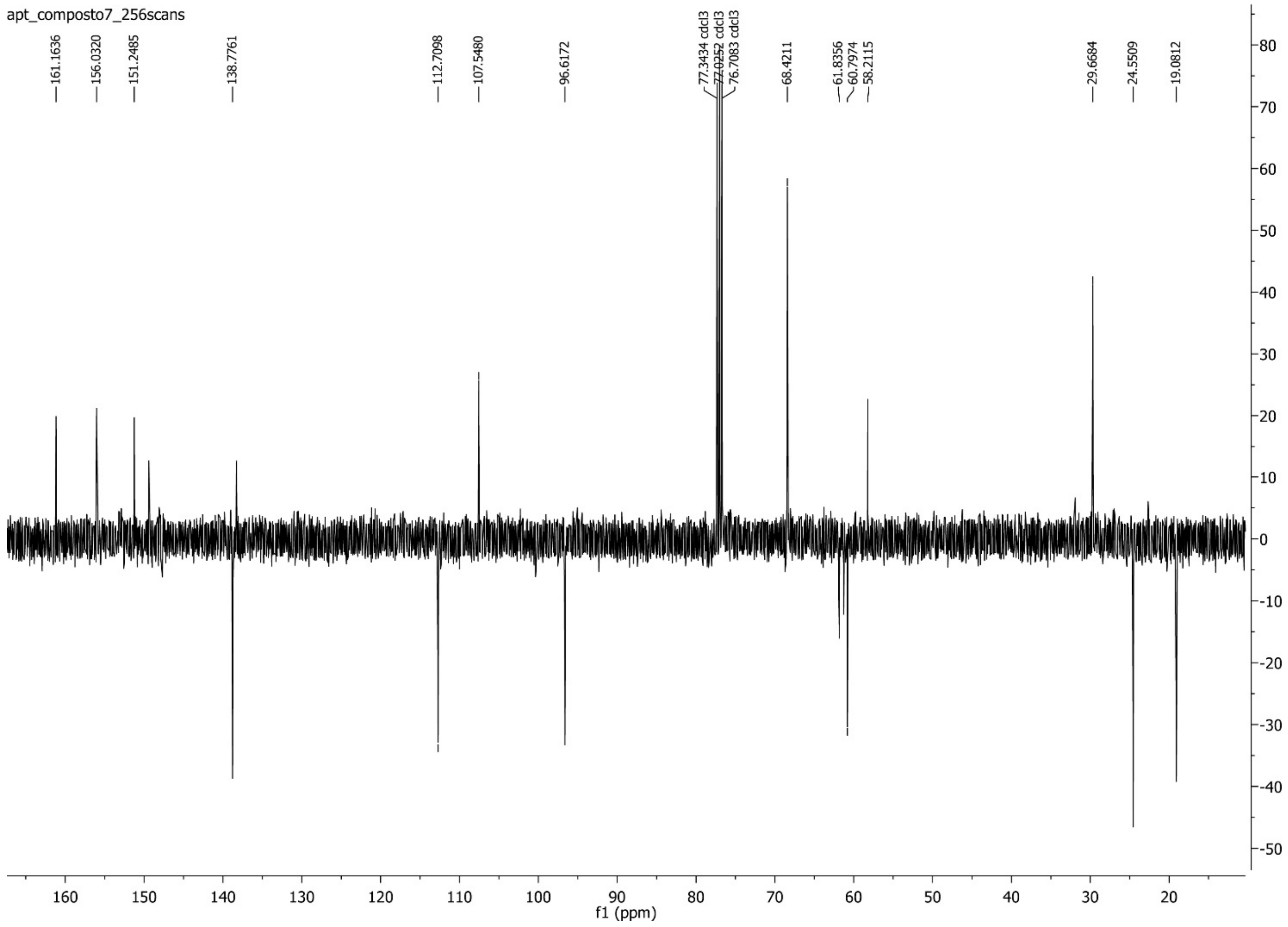

Figure S3. ^{13}C- APT NMR spectrum of 5,6-dimethoxy-7-(2′,3′-epoxy-3′-methylbutyloxy) coumarin (**7**) ($CDCl_3$, 100 MHz).

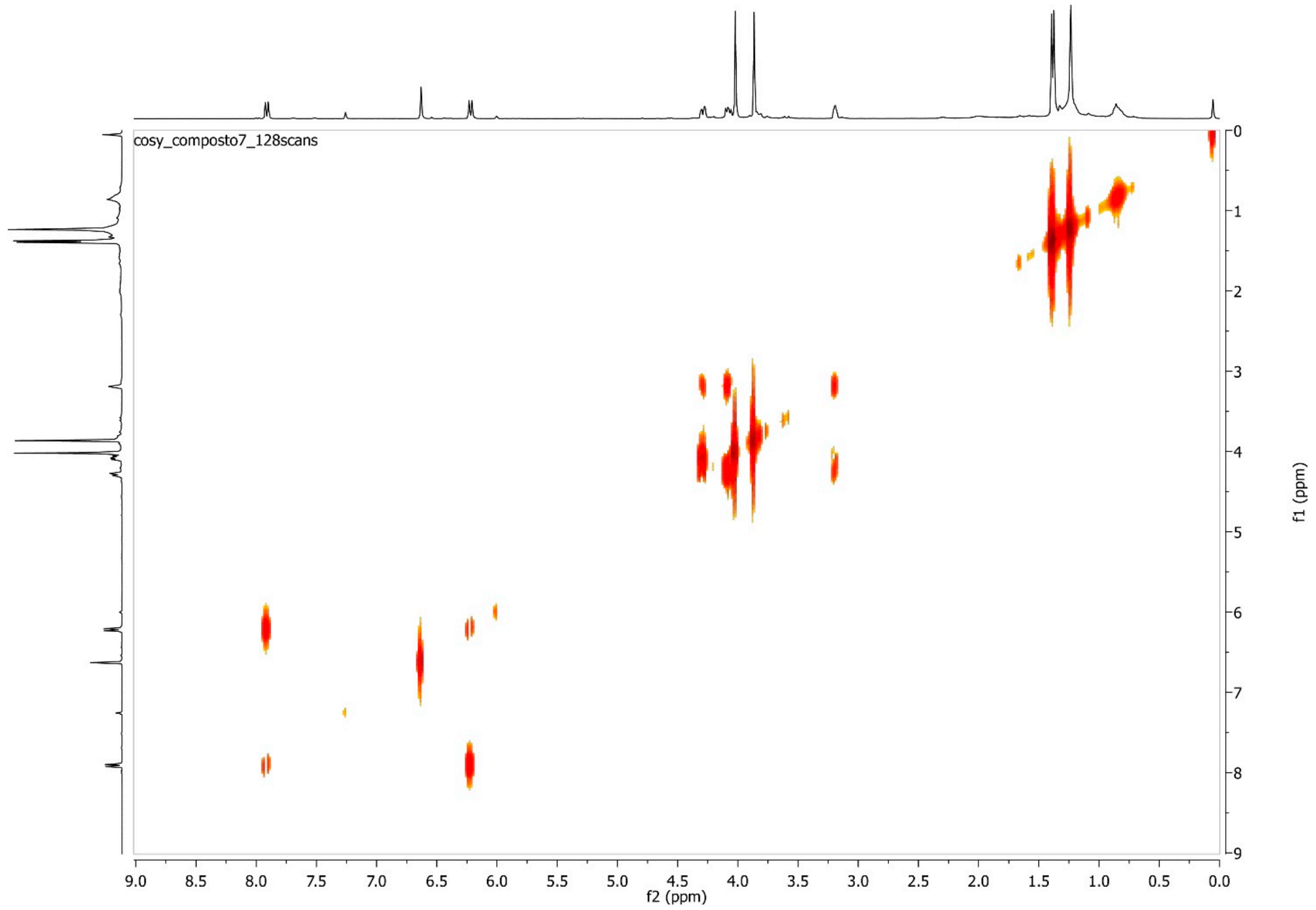

Figure S4. ^{1}H-^{1}H COSY NMR spectrum of 5,6-dimethoxy-7-(2′,3′-epoxy-3′-methylbutyloxy) coumarin (**7**) ($CDCl_3$, 400 MHz).

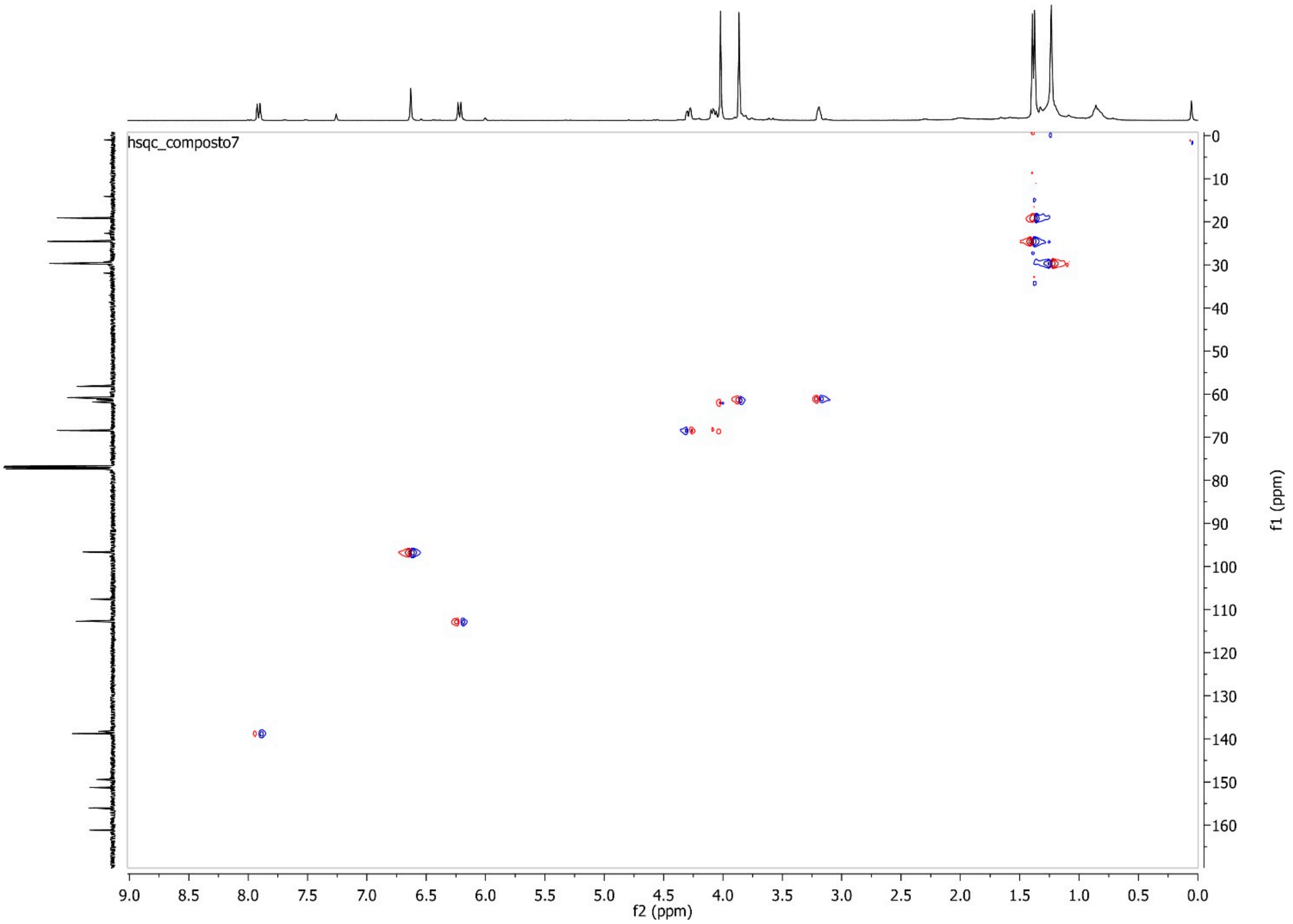

Figure S5. HSQC NMR spectrum of 5,6-dimethoxy-7-(2′,3′-epoxy-3′-methylbutyloxy) coumarin (**7**) ($CDCl_3$, 400 × 100 MHz).

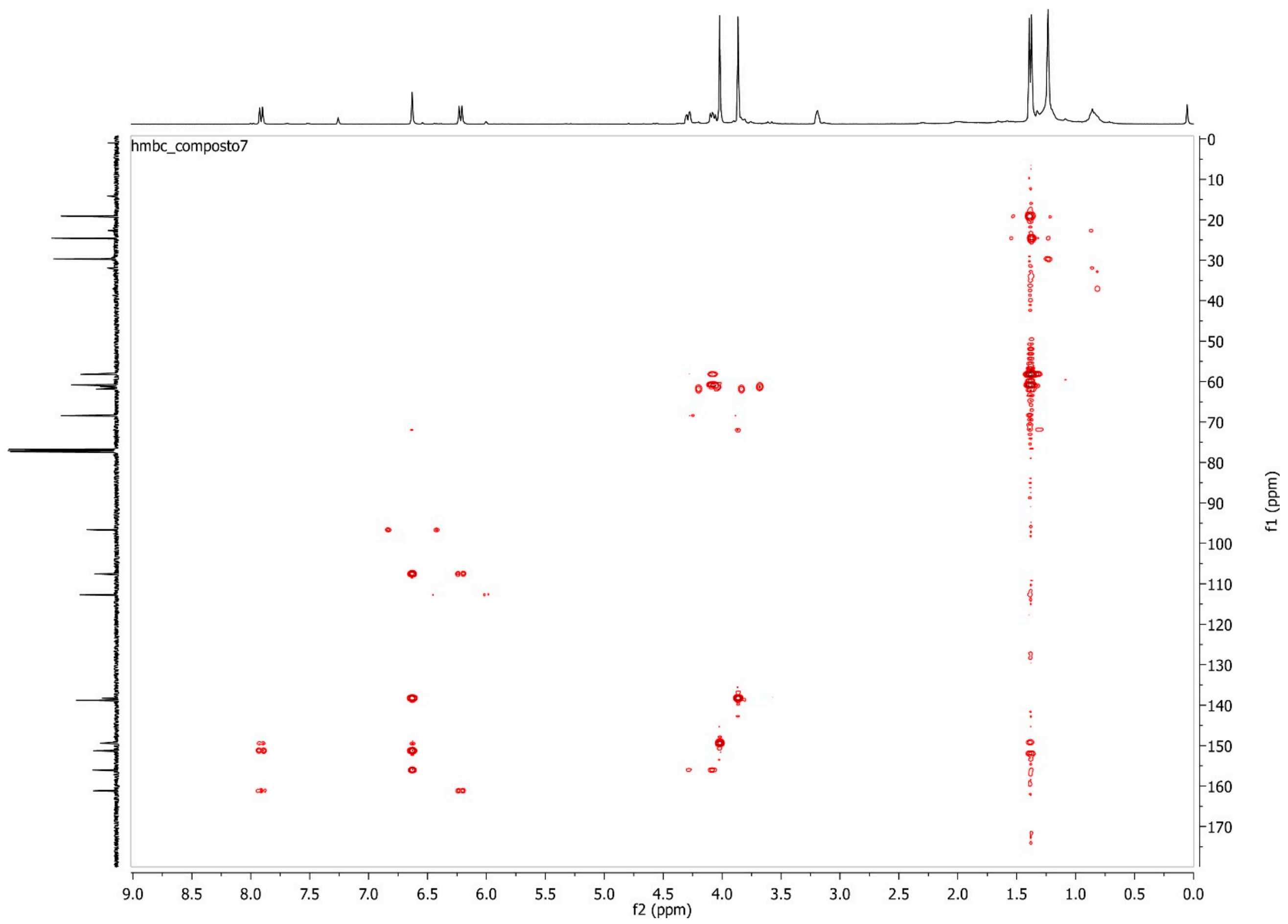

Figure S6. HMBC NMR spectrum of 5,6-dimethoxy-7-(2′,3′-epoxy-3′-methylbutyloxy) coumarin (**7**) ($CDCl_3$, 400 × 100 MHz).

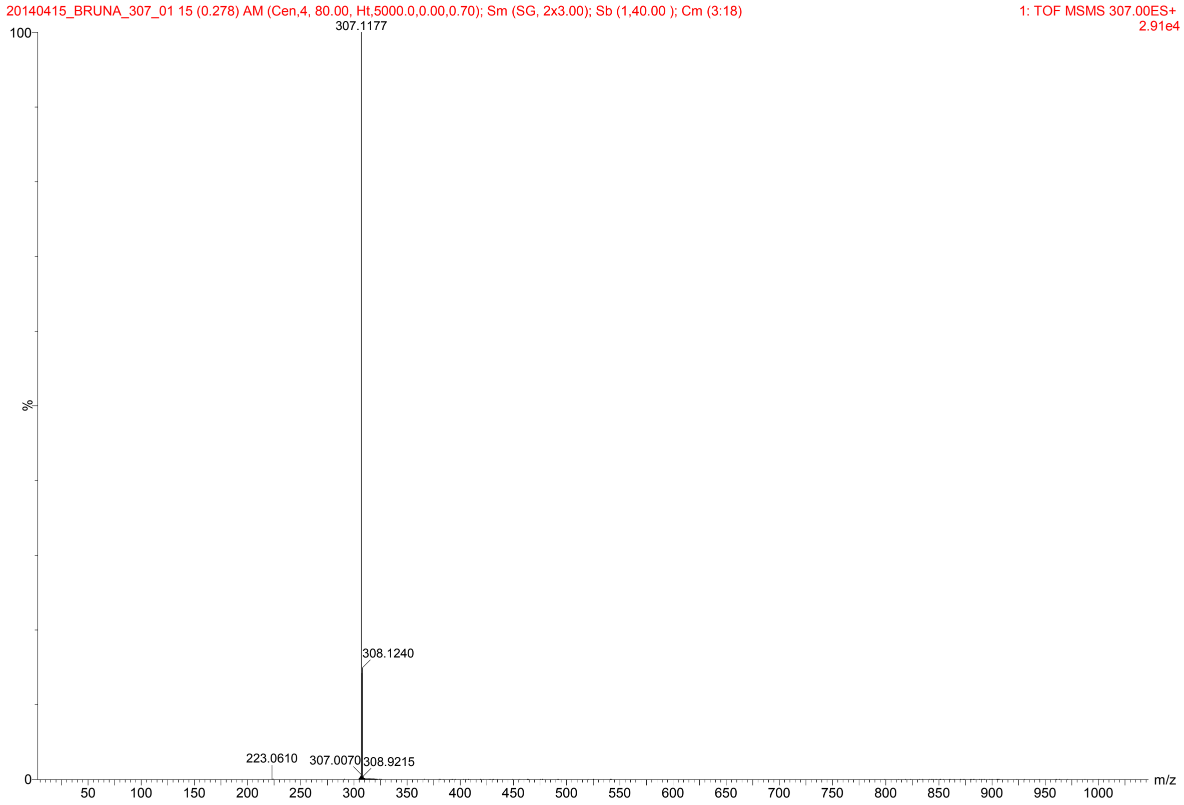

Figure S7. MS/MS spectrum of 5,6-dimethoxy-7-(2′,3′-epoxy-3′-methylbutyloxy)coumarin (**7**).

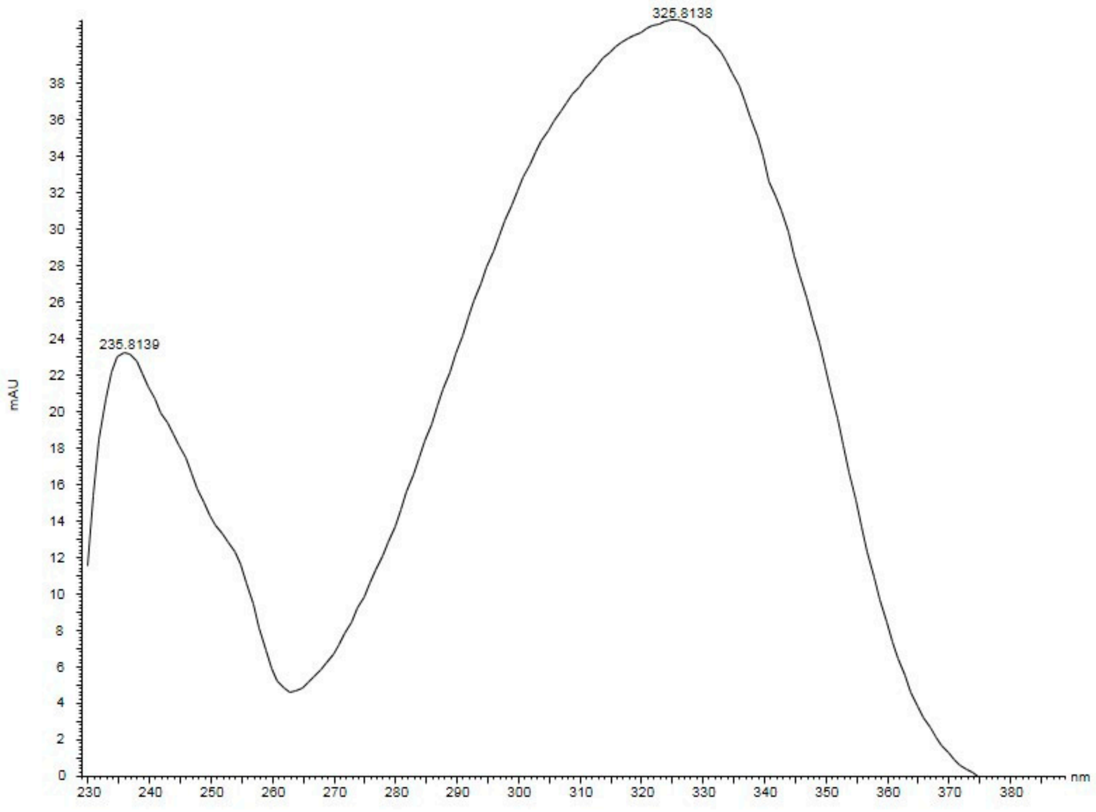

Figure S8. UV spectrum of 5,6-dimethoxy-7-(2′,3′-epoxy-3′-methylbutyloxy)coumarin (**7**).

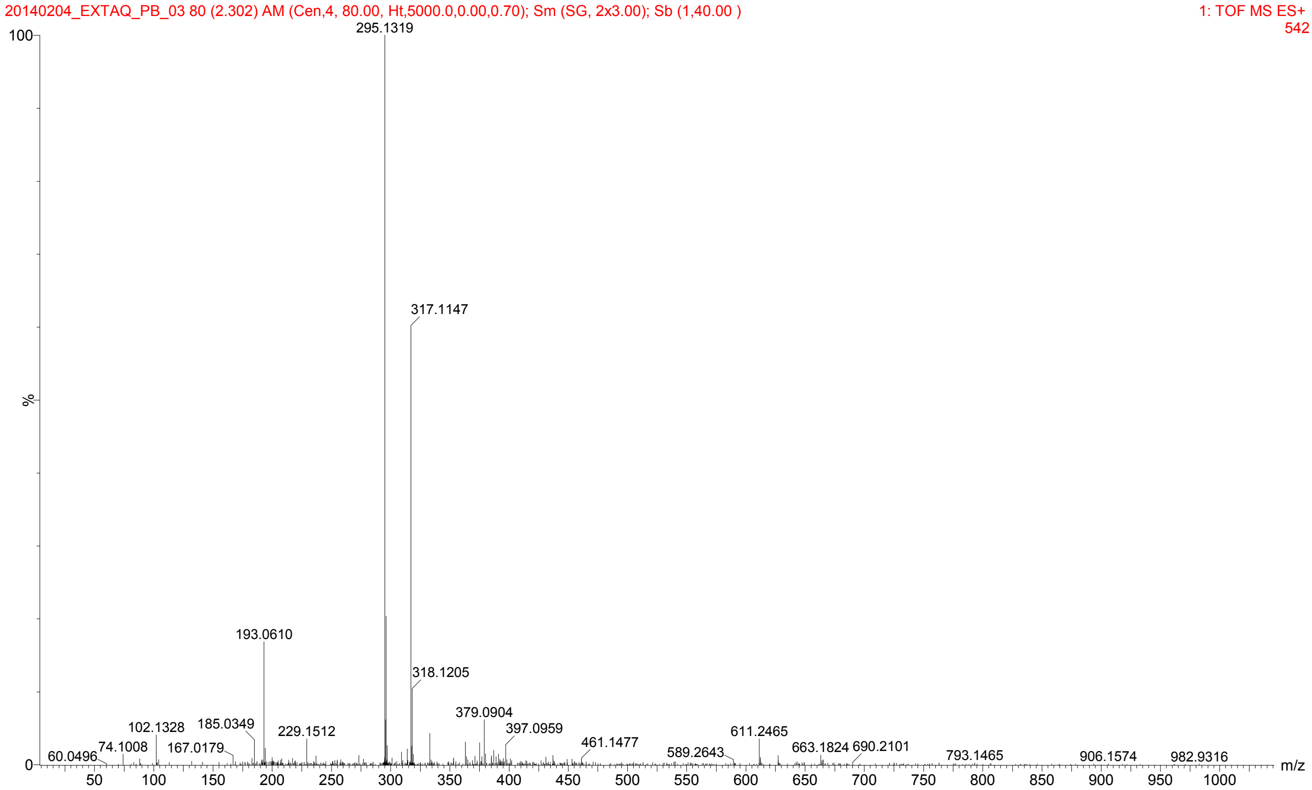

Figure S9. ESI-MS spectrum of 7-(2′,3′-dihydroxy-3′-methylbutyloxy)-6-methoxycoumarin (**1**).

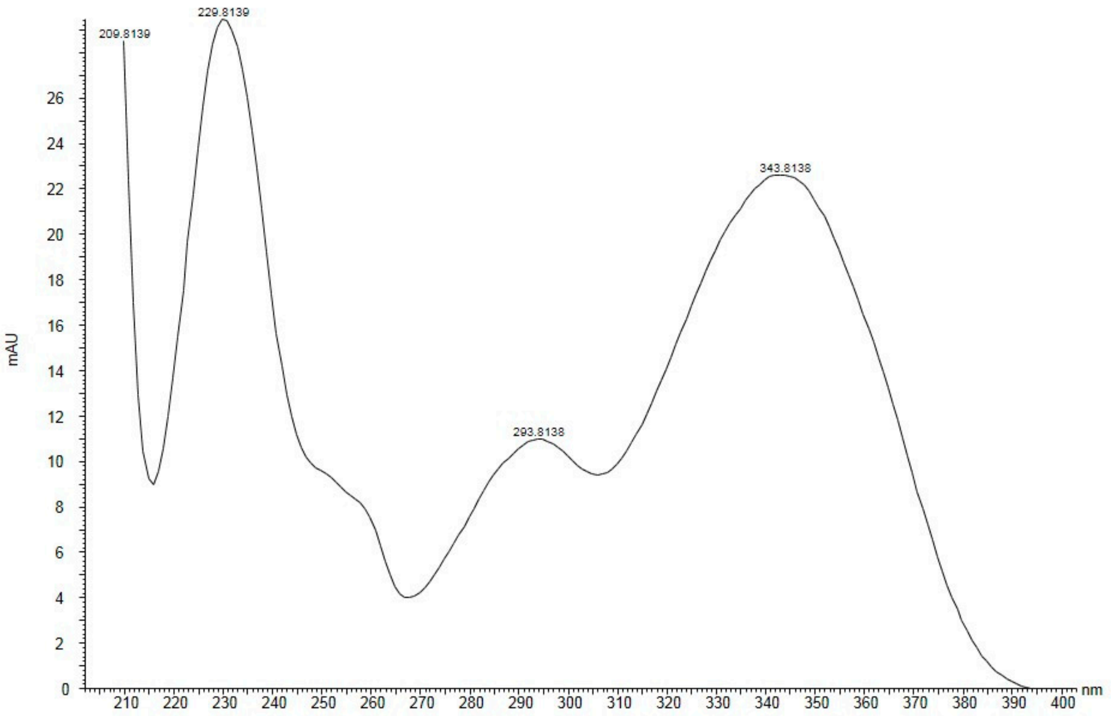

Figure S10. UV spectrum of 7-(2′,3′-dihydroxy-3′-methylbutyloxy)-6-methoxycoumarin (**1**).

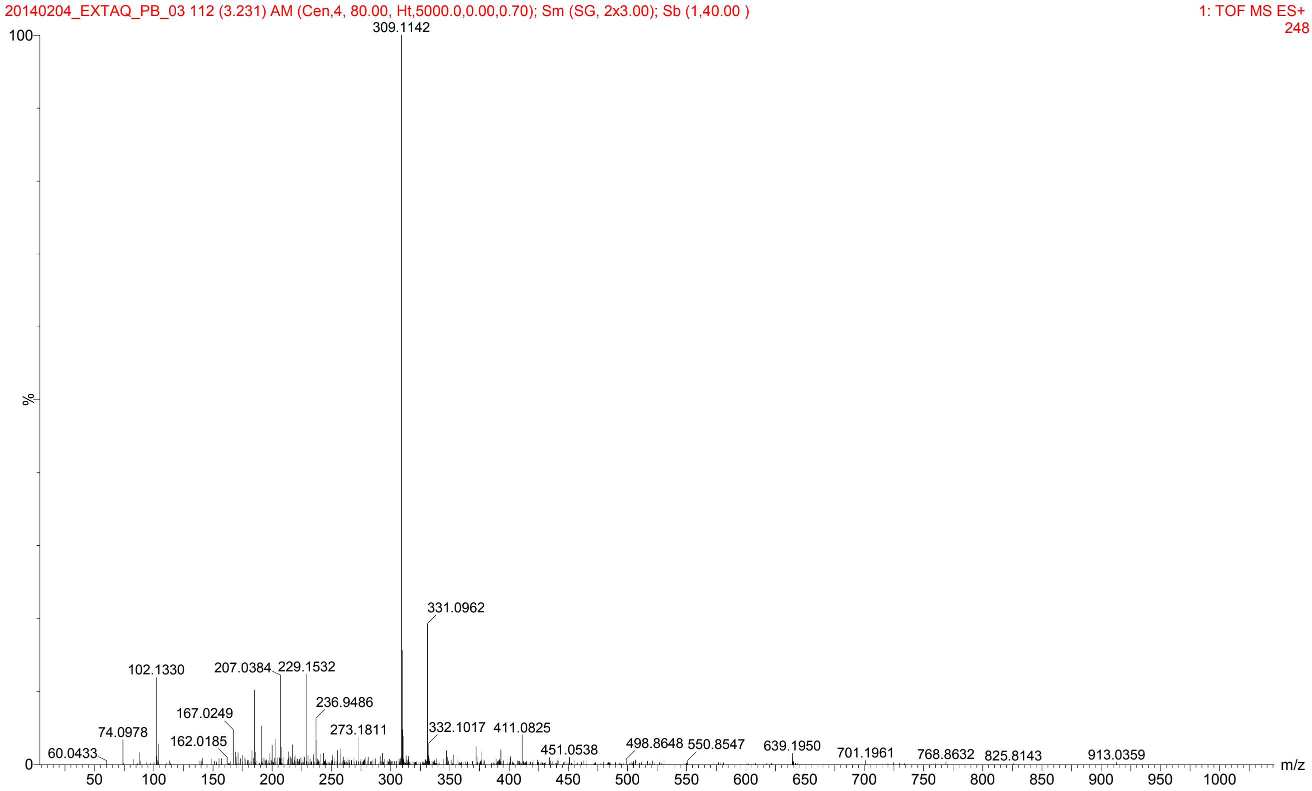

Figure S11. ESI-MS spectrum of 5-(2′,3′-dihydroxy-3′- methylbutyloxy)-6,7-methylenedioxycoumarin (**2**).

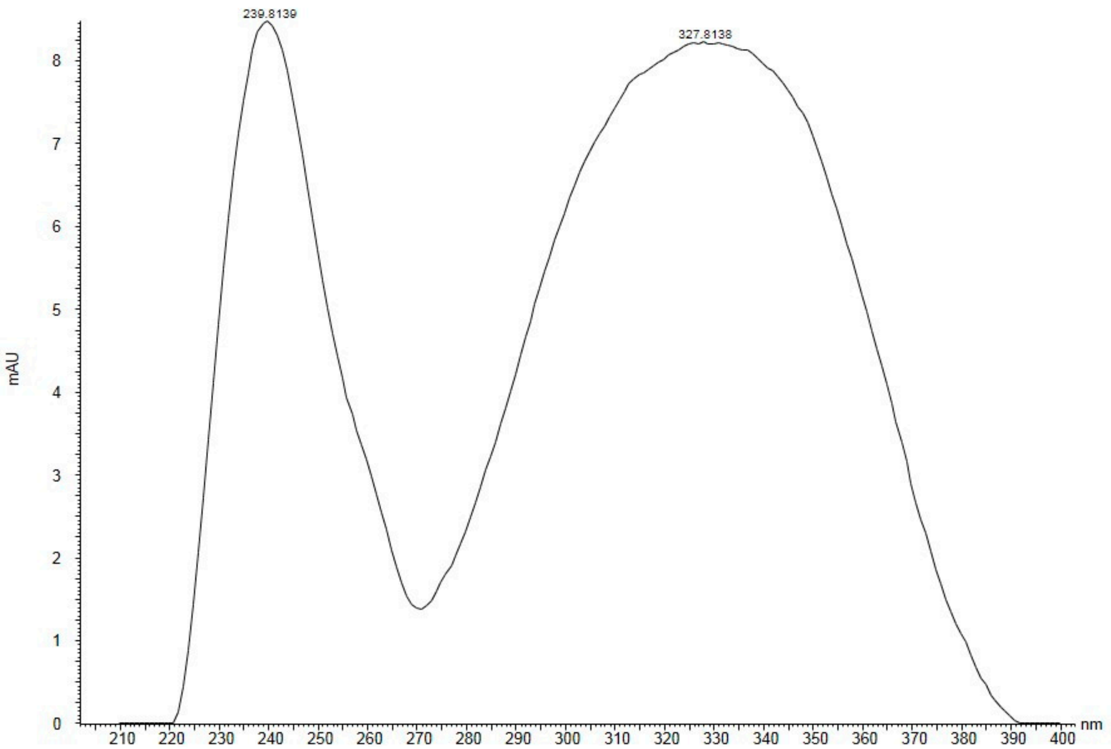

Figure S12. UV spectrum of 5-(2′,3′-dihydroxy-3′- methylbutyloxy)-6,7-methylenedioxycoumarin (**2**).

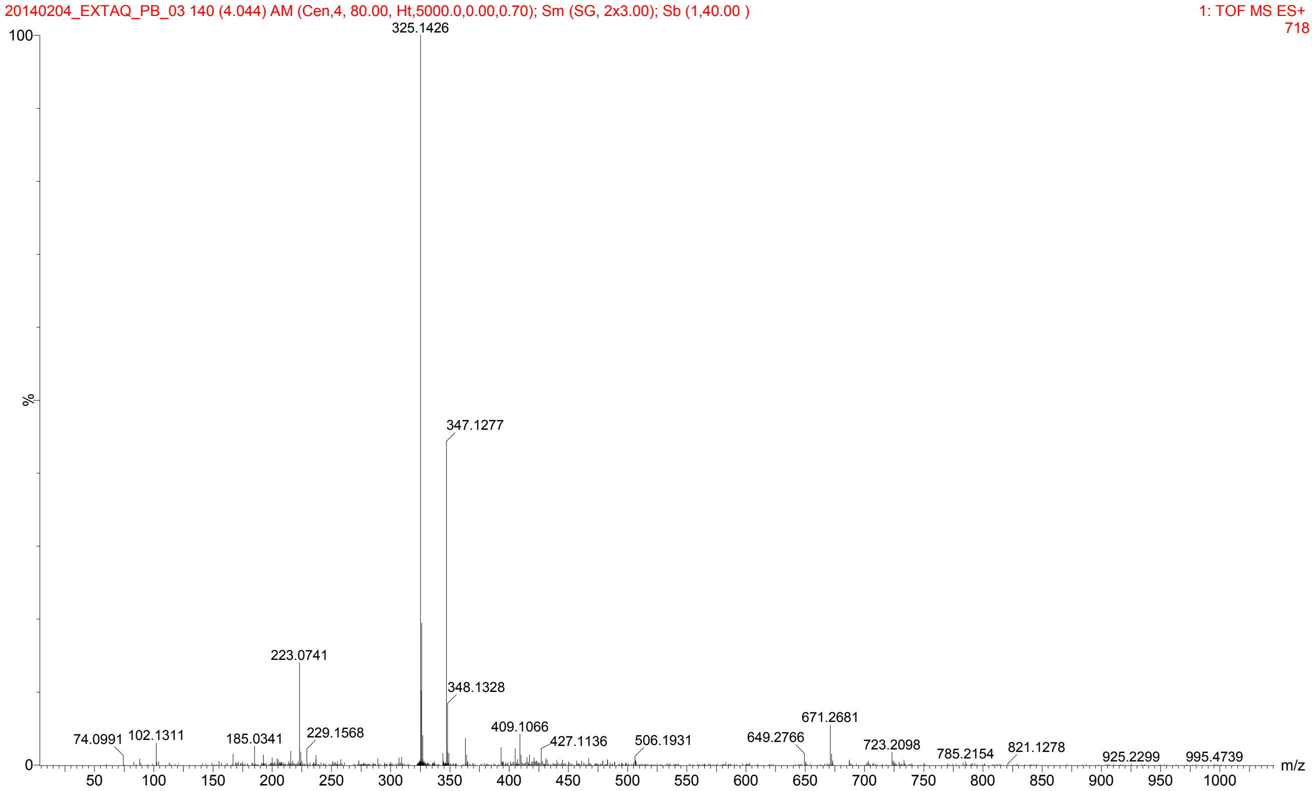

Figure S13. ESI-MS spectrum of 5,6-dimethoxy-7-(3′-methyl-2′,3′-dihydroxybutyloxy) coumarin (**3**).

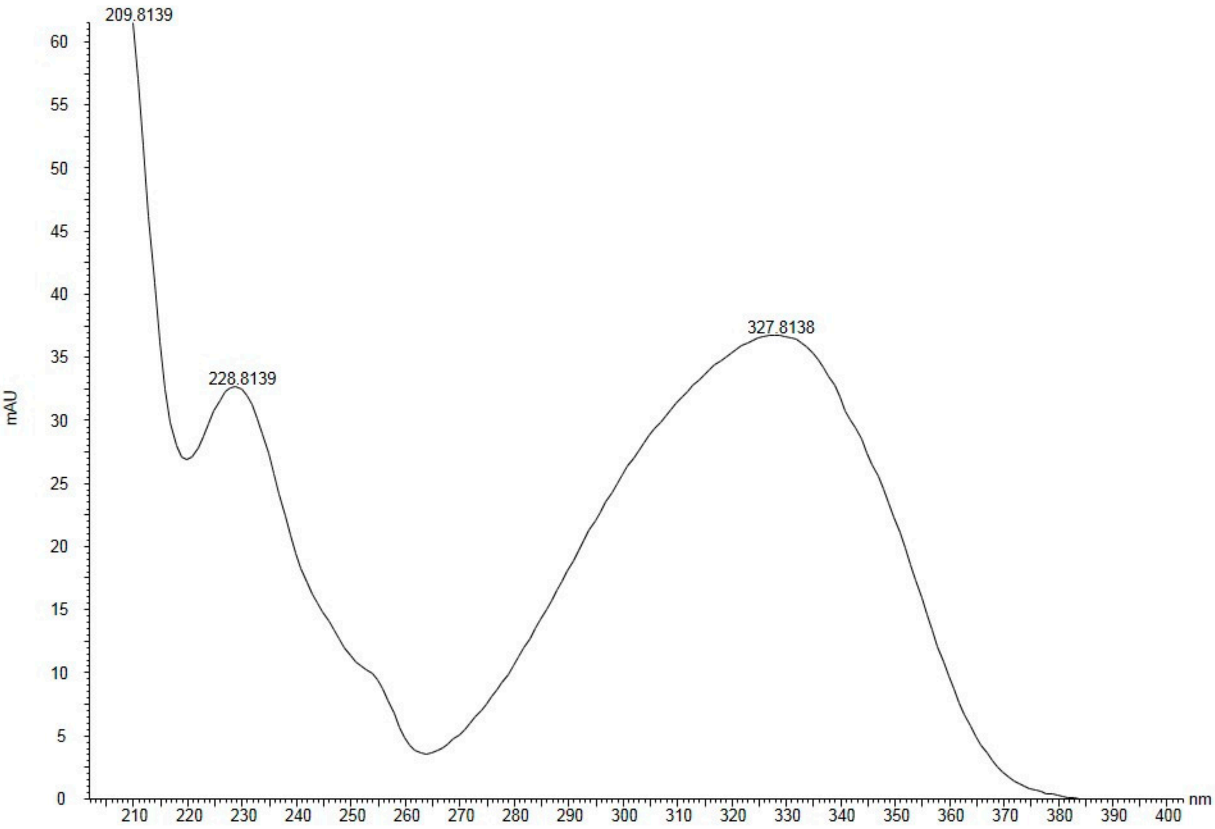

Figure S14. UV spectrum of 5,6-dimethoxy-7-(3′-methyl-2′,3′-dihydroxybutyloxy) coumarin (**3**).

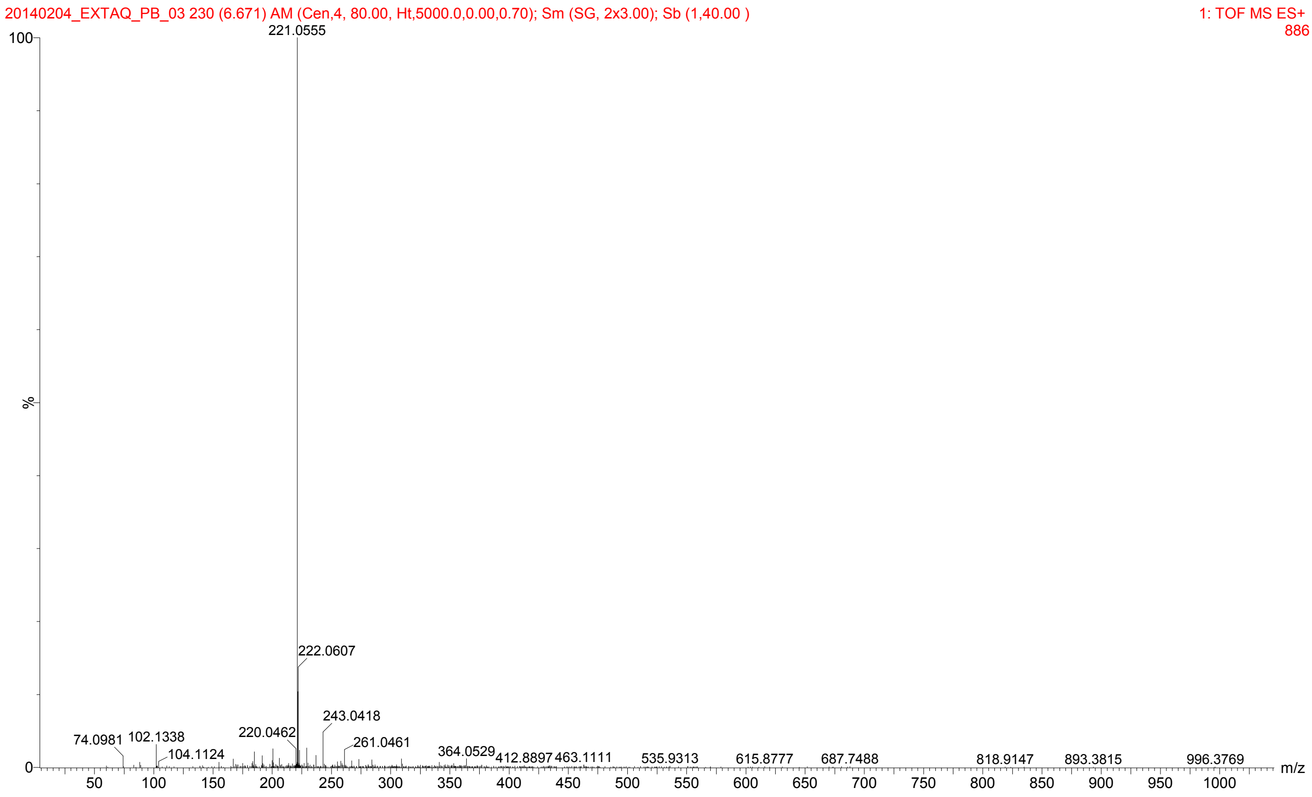

Figure S15. ESI-MS spectrum of 5-methoxy-6,7-methylenedioxycoumarin (**4**).

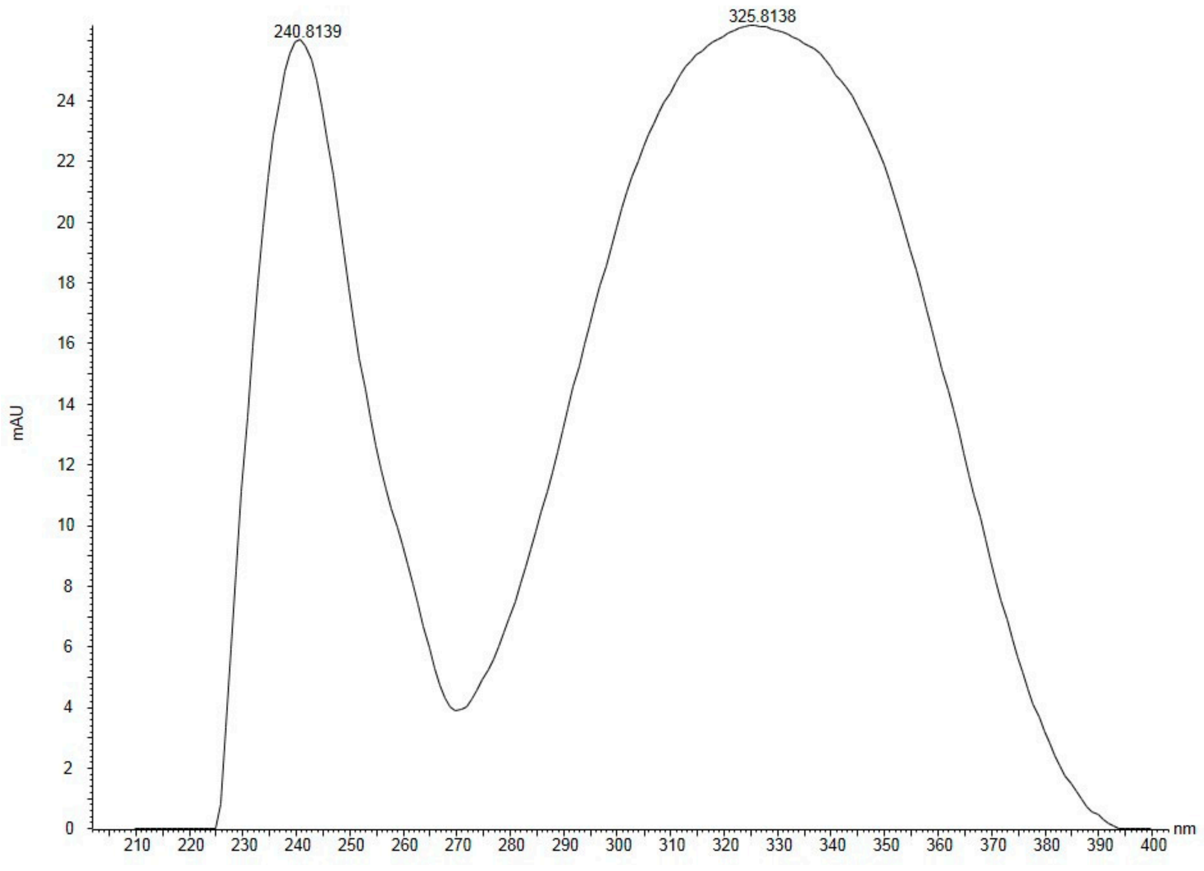

Figure S16. UV spectrum of 5-methoxy-6,7-methylenedioxycoumarin (**4**).

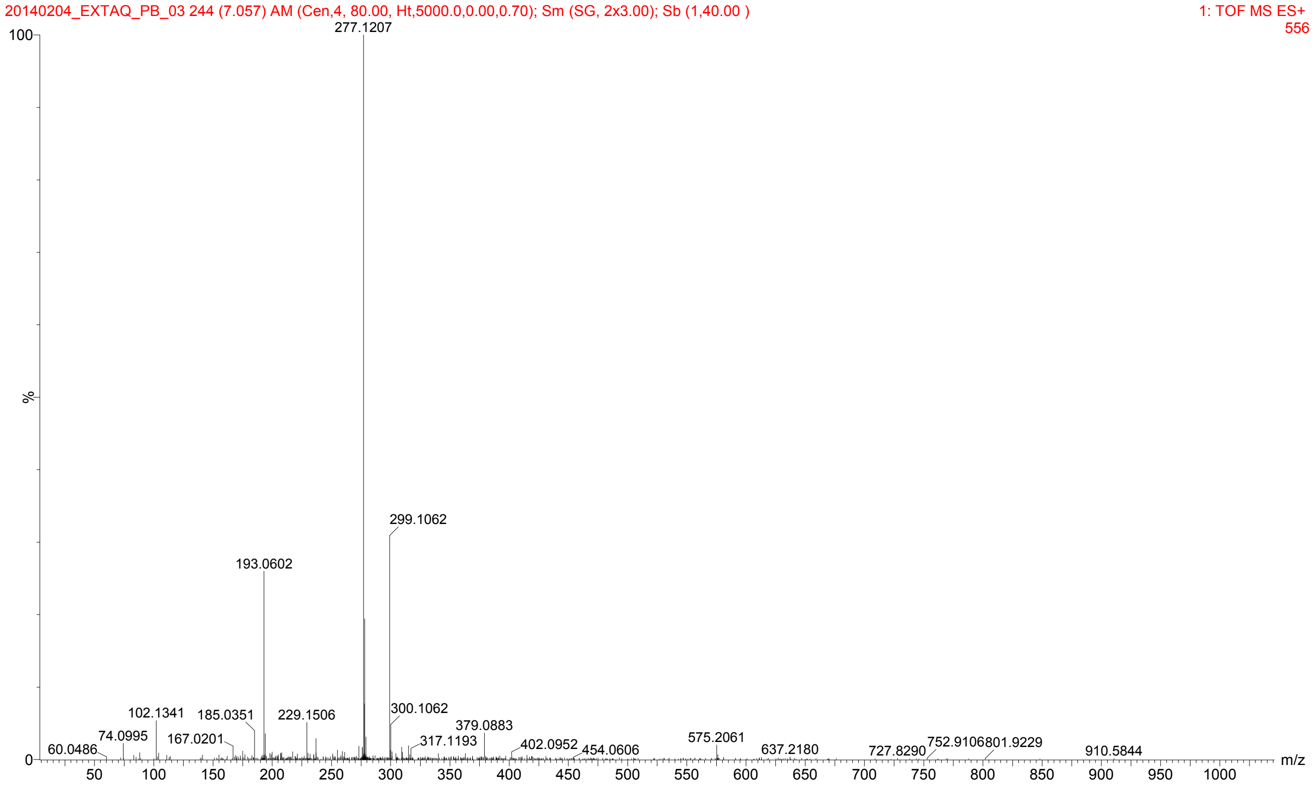

Figure S17. ESI-MS spectrum of 7-(2′,3′-epoxy-3′-methyl-3′-butyloxy)-6-methoxycoumarin (**5**).

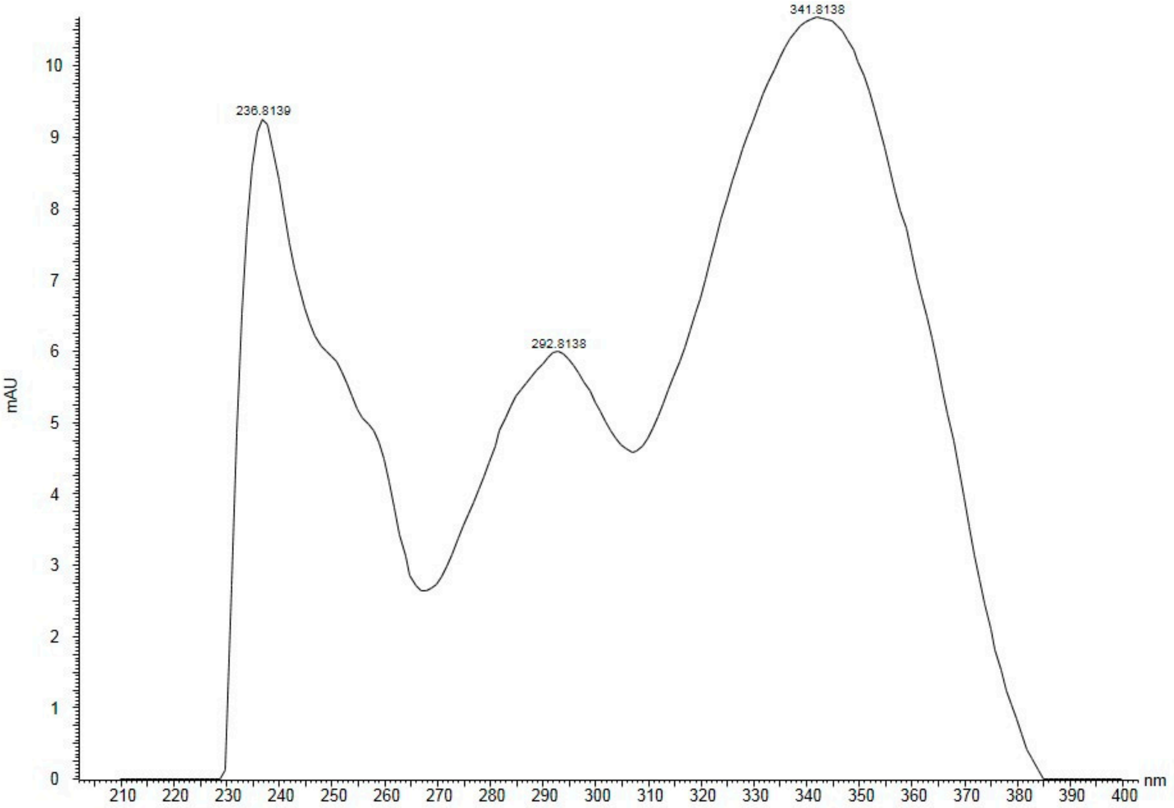

Figure S18. UV spectrum of 7-(2′,3′-epoxy-3′-methyl-3′-butyloxy)-6-methoxycoumarin (**5**).

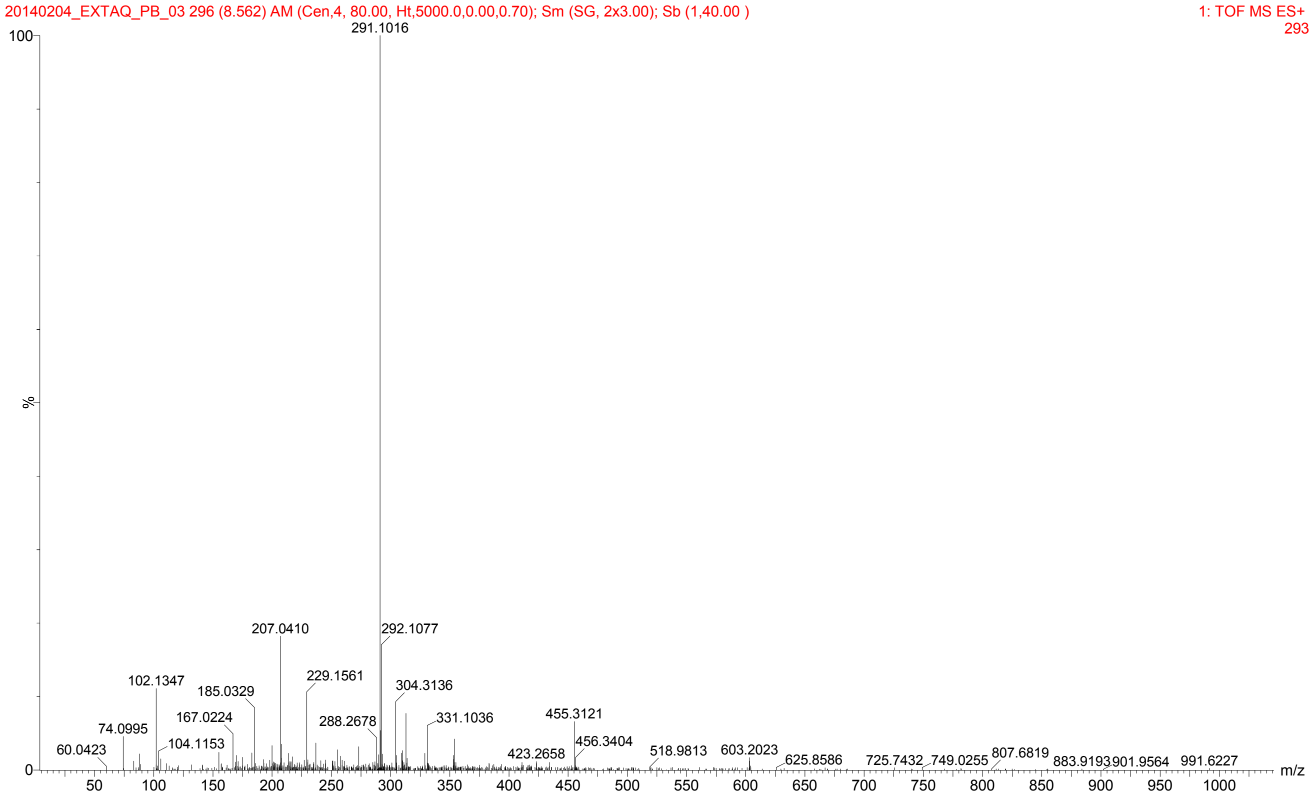

Figure S19. ESI-MS spectrum of 5-(2′,3′-epoxy-3′- methylbutyloxy)-6,7-methylenedioxycoumarin (**6**).

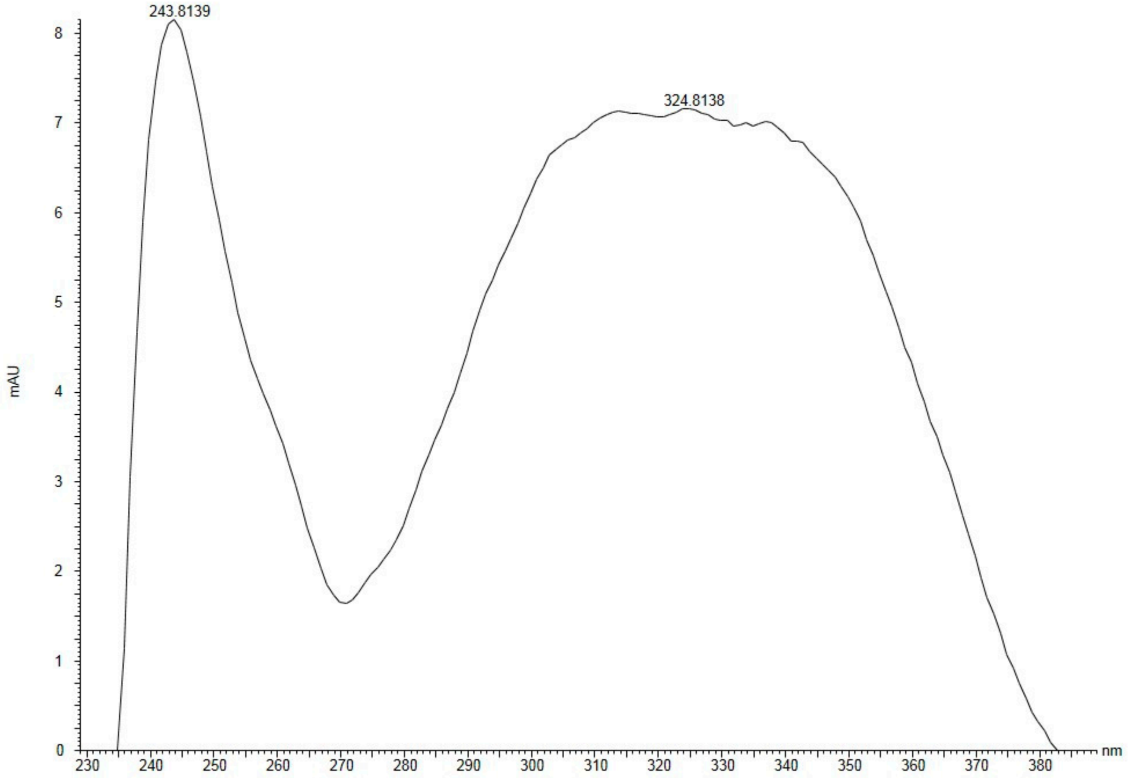

Figure S20. UV spectrum of 5-(2′,3′-epoxy-3′-methylbutyloxy)-6,7-methylenedioxycoumarin (**6**).
